# Seasonal and diel influences on bottlenose dolphin acoustic detection determined by whistles in a coastal lagoon in the southwestern Gulf of California

**DOI:** 10.7717/peerj.13246

**Published:** 2022-05-18

**Authors:** Marco F. W. Gauger, Eduardo Romero-Vivas, Myron A. Peck, Eduardo F. Balart, Javier Caraveo-Patiño

**Affiliations:** 1 Centro de Investigaciones Biológicas del Noroeste S.C., La Paz, Baja California Sur, México; 2 The Netherlands Royal Institute of Sea Research, Den Burg, Texel, Netherlands

**Keywords:** Passive acoustic monitoring, Modelling presence, Hierarchical GAM, Moon phase, Semidiurnal tide, Semi-enclosed lagoon, Continuous presence of bottlenose dolphins

## Abstract

Marine mammals in subtropical coastal habitats are sentinels of the health of the ecosystem and offer important ecosystem services. They rely on prey that pursues feeding opportunities, while both avoid unfavorable conditions. In many cases, these predator-prey dynamics fluctuate seasonally and are regulated by lunar, tidal, and/or diel cycles (hour). However, these rhythmical patterns may vary under different seasonal conditions. Bottlenose dolphins (*Tursiops truncatus*) in the Ensenada de La Paz in Baja California Sur, Mexico, were detected acoustically over the course of an annual cycle on 21 separate occasions, covering 640 h from June 2017 to May 2019. The presence of bottlenose dolphins was examined using Generalized Additive Models (GAM) including variables that are related directly to their habitat (direct variables: hour, distance, depth) and to their prey (indirect variables: SST, moon phase and tides). Seasonal differences in the presence of bottlenose dolphins were influenced more by indirect variables (explained deviance: 34.8% *vs*. 37.7%). Hourly acoustic detections occurred less frequently when SST exceeded 27.4 °C (Aug–End of Nov.) and more frequently at moderate temperatures (22.7 °C to 26.3 °C) in May through July. Moreover, bottlenose dolphins were detected more frequently during waning and new moon phases, at the onset of flood and ebb tides, and during day (04:00 to 20:00). The seasonal differences in acoustic detections rates were highlighted by the global GAM and hierarchical clustering. The strong seasonal pattern indicated possible interactions with rhythmic pattern of bottlenose dolphins. Four candidate variables (SST, moon, tide, and hour) were tested for plausible interaction terms additional to their individual consideration, out of which only hour changed significantly between seasons. The patterns of presence likely increase feeding opportunities or may favor other behaviors such as socializing, resting, or nursing. These might prove responsible for the distinct occurrence and hourly patterns of bottlenose dolphins.

## Introduction

Marine mammals, and cetaceans in particular, require specific food to meet their energetic requirements (Spitz et al., 2012), sustain active tissue, and for reproduction (Caraveo-Patiño et al., 2009; Gelippi et al., 2020). To sustain these needs, they migrate periodically between feeding and mating grounds, or remain in areas that can provide high quality food all year long (Huang et al., 2011). Coastal areas as lagoons and estuaries in tropical, subtropical, and temperate regions are known to attract cetaceans for this reason (*e.g*., Salinas Zacarías, 2005; Sheaves et al., 2014), but they can function as well as nursery areas (*e.g*., Marcín-Medina, 2010; Gelippi et al., 2020), provide shelter and protection from predators (*e.g*., Barros & Wells, 1998). Hence, it is imperative to understand and monitor the variables that determine their presence in coastal areas, to ensure the equilibrium of the ecosystem including the changing presence and distribution of their prey.

The spatiotemporal distribution of marine mammals varies according to the environmental characteristics of their habitat and the level of predator-prey interactions (MacLeod et al., 2006; Spitz et al., 2012). Depending on the trophic level of the predator in question, it is relevant if the odontocetes’ presence depends directly, indirectly, or both on these characteristics (Guisan & Zimmermann, 2000; Wells & Scott, 2002). This is especially important in coastal habitats, where odontocetes live in complex food webs (Díaz-Uribe, Arreguín-Sánchez & Cisneros-Mata, 2007) that are difficult to access and to sample quantitatively. They are known to react fast to changing environmental conditions in coastal areas, offer relevant ecosystem services (Arellano-Peralta & Medrano-González, 2015) and are considered sentinels of the seas (Wells et al., 2004; Bossart, 2011).

Recent studies discussed that the presence and distribution patterns of certain odontocetes can modulate rhythmically under different seasonal and tidal conditions (Fernandez-Betelu et al., 2019; Zein et al., 2019) influencing their interaction with prey and selection of predation areas. Spatiotemporal variation in different prey species (Franks, 1992; Gibson, 2003; Reis-Filho, Giarrizzo & Barros, 2016) and seasonal changing productivity can affect fish assemblages, as species adapt to migrating prey, competitors, and predators (Pittman & McAlpine, 2003; Reis-Filho, Giarrizzo & Barros, 2016). Consequently, presence of odontocetes under seasonally changing conditions need to be assessed carefully (Eierman & Connor, 2014; Temple et al., 2016; Sprogis et al., 2016). Compared to other top marine predators, odontocetes are relatively easy to study (Garner et al., 2021), as they aggregate in groups (Oswald, Rankin & Barlow, 2008), emit characteristic sounds (Defense Research and Development Atlantic Darmouth (Canada), 2002; Janik & Sayigh, 2013; Sousa-Lima et al., 2013), and need to breathe air. This requirement affects their resting behavior (Sekiguchi & Kohshima, 2003; Lyamin et al., 2008), but also allows them to choose their time of activity independently of the time of day. Consequently, attempts to generalize findings on visual daytime data alone might result in bias. Hydrophones are especially helpful to record either directional click trains for navigation and foraging, or omnidirectional whistles for communication. This information helps to detect animals at day and night, or when adverse climate conditions hamper visual detections and hence are useful to control for possible data deficits. The high temporal resolution of acoustic data allows differential analysis that can consider seasonal, monthly, tidal, and diel conditions, as well as to test for interactions between these variables (Fernandez-Betelu et al., 2019; Zein et al., 2019). However, the probability of sound detection depends on numerous factors (Au, 1993; Oswald, Rankin & Barlow, 2008; Finneran et al., 2014) what makes a quantitative and/or qualitative estimation a challenge. Furthermore, the technical specifications of these devices and high costs limit their use, except when custom-made options are available (Joy, Hamilton & Babb, 2012; Bustamante, Romero Vivas & Beristain, 2013; Caldas-Morgan, Alvarez-Rosario & Rodrigues Padovese, 2015; Gauger, Caraveo-Patiño & Romero-Vivas, 2021).

**Figure 1:**
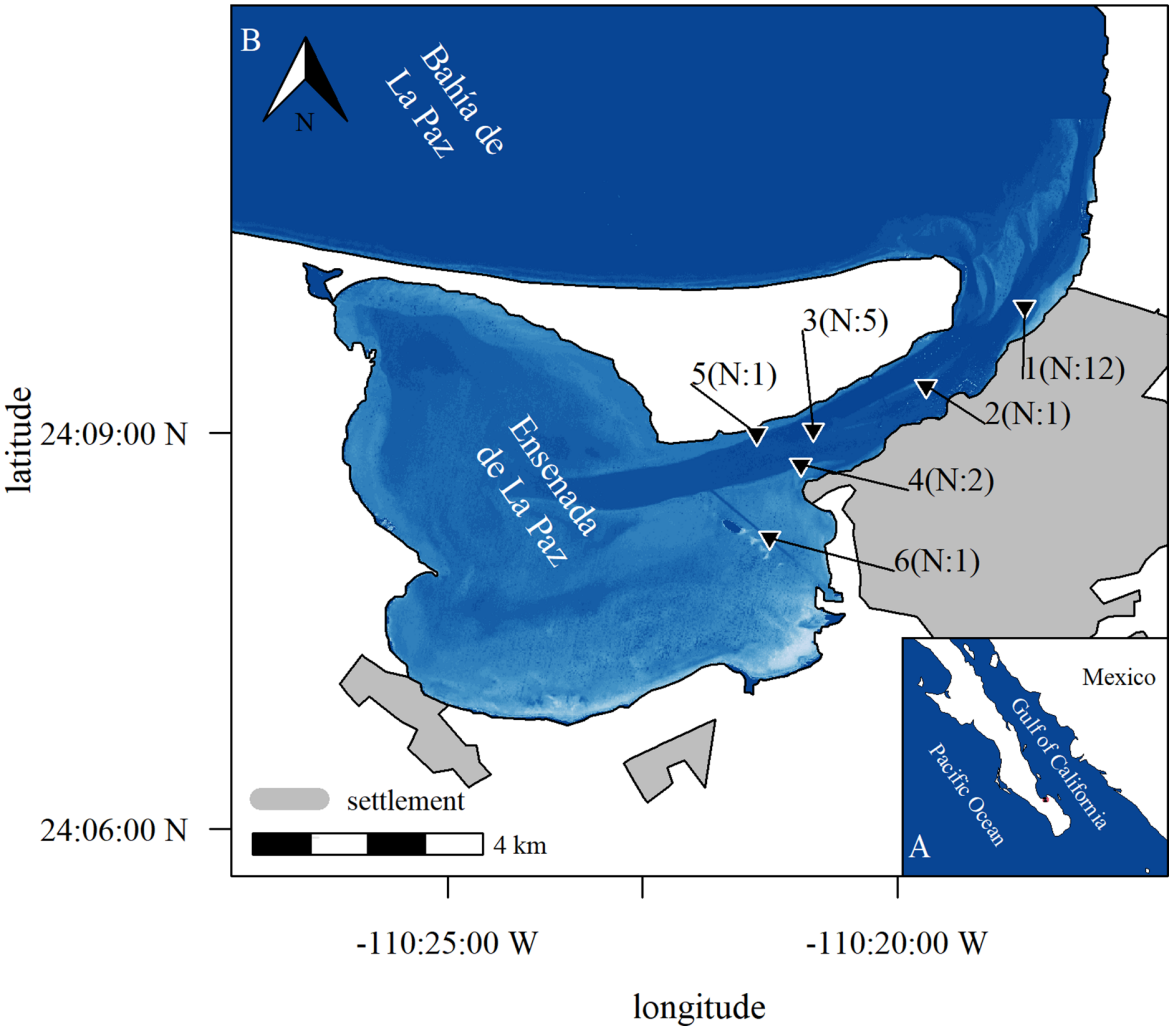
Study area. Study area with position of stations where acoustic equipment was moored in Ensenada de La Paz with number N of deployments (b), Baja California Sur, Mexico (a). Image in subplot a was retrieved from satellite imaginary. It represents relative depth as bathymetric map is missing to highlight features as channel and shallow areas (Copernicus Sentinel 2 data Aug. 15th 2017, preprocessed by European Space Agency; city limits obtained from Comisión Nacional del Agua, Mexico; Datum: WGS 1984, visualization with R software; Bivand, Keitt & Rowlingson, 2021; Hijmans, Van Etten & Hijmans, 2010).

The tidal regime of lagoons is mostly analyzed using factorial states (low tide, high tide, flood, ebb), what is appropriate in most cases that are characterized by a stable tidal regime (Fury & Harrison, 2011). However, such a classification is arbitrary in lagoons along the Northeast Pacific coast, which are characterized by a semi-diurnal mixed tidal regime that changes twice per month from one to two tides per day (Gómez-Valdés, Delgado & Dworak, 2003). Instead, a numerical approach is more appropriate to represent the dynamic nature of the phenomenon, as in the example of the Ensenada de La Paz, Baja California Sur, Mexico (Fig. 1). Previous studies (Acevedo, 1991a, 1991b; Marcín-Medina, 1997, 2010) showed that the only cetacean that frequents this lagoon regularly is the bottlenose dolphin (*Tursiops truncatus*, Montagu, 1821), although other cetacean species occur in the adjacent waters (Flores-Ramírez et al., 1996; Cubero-Pardo, 2007; Salvadeo et al., 2009). Land and boat-based studies showed that bottlenose dolphins are present mostly in small (0–10 animals; 89%) to medium sized groups (12–40 animals; 11%) and enter the lagoon to feed, rest, and nurse their young (Acevedo, 1991a, 1991b; Marcín-Medina, 1997, 2010). The presence and distribution of bottlenose dolphins in the Ensenada de La Paz were reported to change under different seasonal and tidal conditions (Acevedo, 1991b; Marcín-Medina, 1997; Moreno-Zuñiga, 2013). The presented models, however, did not specify what variables acted directly or indirectly on bottlenose dolphins. Moreover, nocturnal and other unfavorable conditions were underrepresented in these studies (Acevedo, 1991a, 1991b; Salinas Zacarías, 2005; Marcín-Medina, 2010). The only available previous study that implemented passive acoustic in this area (Gauger, Caraveo-Patiño & Romero-Vivas, 2021) focused on the continuous presence of bottlenose dolphins during day and night, and indicated higher presence of animals at night. However, it was implemented for a short time only (June–November 2018) and covered seasonal variation only partly and therefore did not allow a generalized analytical approach.

In this work, a passive acoustic monitoring was implemented in the Ensenada de La Paz to test and model different environmental variables that act directly or indirectly on bottlenose dolphins’ presence. Specifically, the study was based on three main hypotheses. First, acoustic detections of bottlenose dolphins in the Ensenada de La Paz fluctuate seasonally. Second, acoustic detections of bottlenose dolphins fluctuate rhythmically and correlate with dynamic and factorial environmental variables. Several factorial (depth, distance from shore), and dynamic (moon phases, tides, and hour) physical processes are indeed known to influence the presence bottlenose dolphins directly or their prey (Gibson, 2003), and consequently, induce rhythmic presence of bottlenose dolphins (Fernandez-Betelu et al., 2019). Third, the fluctuation of acoustic detections of bottlenose dolphins interacts between long (SST) and short-term (moon, tide, and hour) cyclic variables. To test these hypotheses, hydrophones were deployed regularly, relevant variables were evaluated quantitatively, and the ecological space was represented for a complete year.

## Materials and Methods

### Study area

The Ensenada de La Paz is a semi-enclosed lagoon in the southern periphery of Bahía de La Paz, Baja California Sur, Mexico (Fig. 1A). The lagoon is 1–3 m deep, comprises an area of approx. 52.1 km^2^, and connects to the bay by a 6-km long and 1-km wide channel (5 to 10 m deep, Fig. 1B). At the entrance to the lagoon a shallow sandbank limits the direct access to the channel (1.5 m), while two trenches (3–5 m) comprise its main points of entry (Fig. 1B). Earlier studies showed that bottlenose dolphins are the only delphinid species occurring in the lagoon, where they were observed frequently foraging and socializing both in the entrance and in the channel (Acevedo, 1991b; Marcín-Medina, 1997, 2010; Gauger, Caraveo-Patiño & Romero-Vivas, 2021). The shallow depth at the entrance to the lagoon might reduce the probability of recording emissions of bottlenose dolphins beyond the sandbank.

### Data collection

Recording devices were moored periodically during 21 intervals between June 2017 and May 2019. Recorders were deployed at half maximum depth in the channel that connects the Ensenada de La Paz to the Bahía de La Paz (water depth 1.5–5.0 m, Fig. 1B). Stations were chosen at the beginning, the middle, and the end of the channel, to verify the presence of bottlenose dolphins in the entire channel as documented from visual surveys (Acevedo, 1991b; Marcín-Medina, 1997, 2010; Gauger, Caraveo-Patiño & Romero-Vivas, 2021). During each of the 21 deployments one device was moored for up to 48 h at one station. Three times two recorders were deployed simultaneously at two different locations and the interval between recording periods was 6 to 207 days. Recording stations and periods were chosen to represent different seasonal, tidal, and moon phases in different depth ranges and distances from the coast, to obtain relevant information to parametrize an ecological model that explains the presence of bottlenose dolphins (Fig. S1). As more sophisticated devices were not available, custom-made sound recorders (hydrophone sensitivity –193 dB re 1V; Bustamante, Beristain & Romero Vivas, 2017) were deployed (Gauger, Caraveo-Patiño & Romero-Vivas, 2021). The devices recorded continuously for up to 48 h and stored sounds in uncompressed audio files (44.1 kHz, 16-bit, 2 channels, Waveform Audio (WAV) format).

Bottlenose dolphins emit echolocation clicks continuously in the frequency range of 60 to 200 kHz while foraging and for orientation (Au, Benoit-Bird & Kastelein, 2007). The frequency dependent absorption of sound in seawater (Francois & Garrison, 1982) reduces the propagation of clicks to several hundred meters (Jensen et al., 2009). The probability of sound detection is reduced additionally as these sounds propagate primarily in a cone of approx. 20°, beyond which the sound pressure level of click sounds drops by 30 dB or more (Au, 1993; Finneran et al., 2014). On the other hand, whistles are emitted in a narrow-band frequency range of 1.2–35 kHz that are used only for communication (Janik & Sayigh, 2013). The repetition rate of whistles depends on several aspects as the behavior, number of animals, and presence of other species causing animals to be not communicating but echolocating, or emitting whistles repeatably (Oswald, Rankin & Barlow, 2008). Whistles spread omnidirectional and have a theoretical range of several kilometers due to the relatively low absorption in this frequency range (Jensen et al., 2012). Here, despite a lower probability of detection due to their lower repetition rate but a higher theoretical detection range due to lower frequency dependent absorption, recordings focused only on whistles to assess the presence of bottlenose dolphins. Even though the sample rate of the recordings is lower than the Nyquist frequency, clicks and buzzes can be detected by their low-frequency components. However, these sounds were discarded from the analysis.

Whistles, as indicators of the bottlenose dolphin presence (Goold, 2000; Hodge et al., 2013; Gregorietti et al., 2021), were detected visually by analyzing audio files in the frequency domain (Adobe Audition, version 3.0, spectrogram settings: 512 points fast Fourier transformed (FFT), Hanning window 50% overlap, dynamic range −110 to 180 dB, display frame duration 4.0 s). In a prior study that used a subset of the data presented here (Gauger, Caraveo-Patiño & Romero-Vivas, 2021) whistles differed significantly between deployments, because whistles were several orders of magnitude more frequent in June and July than in September till November. Following the approach used in click trains of harbor porpoises (Fernandez-Betelu et al., 2019; Zein et al., 2019), detected whistles were pooled if the time between them was smaller than 10 min. Here, the presence of bottlenose dolphins was compared by using the number of intervals per hour with at least one detected whistle: detection positive 10-min intervals per hour (dp10m h^−1^, Data S1).

### Statistical analysis

Exploratory data analyses indicated that acoustic detections (dp10m h^−1^) deviated from normality (Anderson-Darling test: *p* < 0.001) and homogeneity of variances (Levene test: deployments: *p* < 0.001; months: *p* < 0.001).

To test the first hypothesis, acoustic detections of bottlenose dolphins in the Ensenada de La Paz fluctuate seasonally, Kruskal–Wallis test was applied to evaluate differences between deployments and months. Posteriorly, when tests were significant Nemenyi’s all-pairs rank comparison tested for group specific differences (Pohlert, 2021).

To test the second hypothesis, acoustic detections of bottlenose dolphins fluctuate rhythmically and correlate with cyclic environmental variables, cyclic variables (hour and moon phase) were tested for the deviation from uniformity (Hermans-Rasson test; Landler, Ruxton & Malkemper, 2019). This test is more suitable for multimodal situations as in this study (21 deployments) than other tests that assume a von Mises distribution. These cyclic variables were visualized additionally with CircSiZer maps (Lê, Josse & Husson, 2008).

Thereafter, acoustic detections (dp10m h^−1^) were modeled as a function of different temporal, oceanographic, lunar, and biological variables. These were classified as variables that affect primarily prey species (indirect), bottlenose dolphins (direct), or depended on the recording from a technical point of view (technical, Table 1) following the recommendation for ecological modelling (Guisan & Zimmermann, 2000; Guisan, Edwards & Hastie, 2002). Generalized additive models (GAM) were chosen as modeling framework (Hastie & Tibshirani, 1990) as the relationship between descriptive and response variables is most probably nonlinear. Environmental information was obtained from satellite data (Simons, 2019), the software Mar version 10 (http://predmar.cicese.mx/) and different R packages (Table 1). GAMs were fitted assuming a Poisson error structure and applying a log link function (Wood, 2012). A Spearman rank test was performed during variable selection, as GAMs are sensitive to collinearity (Zuur, Ieno & Elphick, 2010; Dormann et al., 2013). If selected variables were correlated (*p* < −0.7 or ρ > 0.7) only one variable was considered in the further selection process. Variable selection followed a forward stepwise selection procedure while using the Akaike Information Criterion (AIC) and AIC weights (Anderson, Burnham & White, 1998).

**Table 1:** Variables considered in the modeling of detection and non-detection.

Variable	Description	Type	Range	Source
Moon phase^i^	Moon phase 0 and 1.0 new moon, 0.5 full moon	Numeric	0–1	Lazaridis, 2014
Tide^i^	Tidal height in m	Numeric	−0.92 to 0.81	http://redmar.cicese.mx/emmc/DATA/LPAZ/MIN/ 04.06.2019
Flow^i^	Change of tidal height in the last 60 min	Numeric	−0.043 to 0.058	
Derivate of tide^i^	Slope of first derivate of cubic function of tidal change of height over last three hours	Numeric	−0.0033 to 0.0037	
Hour^d^	Hour of the day	Categoric	0–24	
Daylength	Time in hours between civil dawn and civil dusk	Numeric	0–24	Thieurmel & Elmarhraoui, 2019
SST^i^	Multi-scale Ultra-high Resolution (MUR) SST in the Ensenada resolution: daily, 0.01°	Numeric	19.6–30.4	https://coastwatch.pfeg.noaa.gov/erddap/griddap/jplMURSST41.html 07.03.2020
SST_BLAP^i^	Multi-scale Ultra-high Resolution (MUR) SST in the Bahía de La Paz resolution: daily, 0.01°	Numeric	21.03–29.90	
Delta_SST^i^	Difference between SST and SST_BLAB	Numeric	−0.41 to 0.66	
Chl^i^	Chlorophyll (mg m-3] NOAA Visible Infrared Imaging Radiometer Suite, 750 m resolution,	Numeric	0.21–82.7	https://coastwatch.pfeg.noaa.gov/erddap/griddap/erdVHNchla1day.html 07.03.2020
Coast^d^	Distance to the coast (km)	Numeric	0.1–1.1	
Distance^d^	Distance to the entrance of the lagoon (km)	Numeric	1.0–7.0	
Mangrove^i^	Distance to nearest mangrove (km)	Numeric	0.1–3.5	
Depth^d^	Depth (m)	Numeric	1.5–5.0	Plumb line at high tide
Bottom^i^	Bottom type (sand, mud, rubble)	Categoric	1–3	Visual observation
Effort^t^	Recording per hour	Numeric	1–60	Start and end of recording not at beginning of hour

**Note:**

Variables considered in the modeling of detection and non-detection of bottlenose dolphins in the Ensenada de La Paz: i, indirect variable; d, direct variable; t, technical variables.

To test the third hypothesis, the fluctuation of acoustic detections of bottlenose dolphins interacts between long (SST) and short-term (moon, tide, and hour) cyclic variables, deployments with similar environmental conditions were pooled by hierarchical clustering on principal components (Lê, Josse & Husson, 2008) and tested for difference in acoustic detections among clusters. Therefore, environmental variables (chlorophyll, sea surface temperature, depth, distance from coast, daylength between dawn and dusk) used in the principal component analysis (PCA) were selected by applying a Kaiser–Meyer–Olkin Measure of Sampling Adequacy test (KMO > 0.5, Nijs, 2021). The principal components that explained more than 70% of the data were considered then to build a hierarchical dendrogram computed from a distance matrix (Ward’s method). Then, the deployments were separated by the hierarchical k-means algorithm into different clusters as proposed by the dendrogram structure (Husson, Josse & Pages, 2010). The acoustic detections among clusters were compared thereafter as described earlier for deployments and months.

To specify possible interactions between seasonal and other cyclic variables, nested versions of the global model were run to test, if the variable effect on bottlenose dolphin presence diverged under different environmental conditions (Pedersen et al., 2019). By grouping variables by the previously defined clusters, it was possible to compare the global model (G) to four nested models. The global and nested models differed in the freedom granted to the smoothing penalty (S: shared penalty, I: individual penalties, GS: general and shared penalties, and GI: general and individual penalties). Model S grouped the variables using a specific basis type (bs, factor.smooth.interaction). This basis allows for a separate smooth considering a factorial variables (*i.e*., cluster), however, kept the smoothing penalty low by estimating only one parameter (s: shared penalty). Model I used for each group an individual smooth penalty (i: individual penalty; Pedersen et al., 2019). Model GS and GI had an additional global smooth for the seasonal variable, to test if a global smooth added information to the model. The parameterization differed, between the two models as the general smooth of model GI was penalized stronger than the individual smooths. This was achieved by penalizing the squared first derivate of the smoothing function instead of the second derivate (see, Pedersen et al., 2019). Tested variables included SST, moon, tide and derivate of tide, and hour, which were grouped by the clusters, obtained from HCPC analysis.

**Figure 2:**
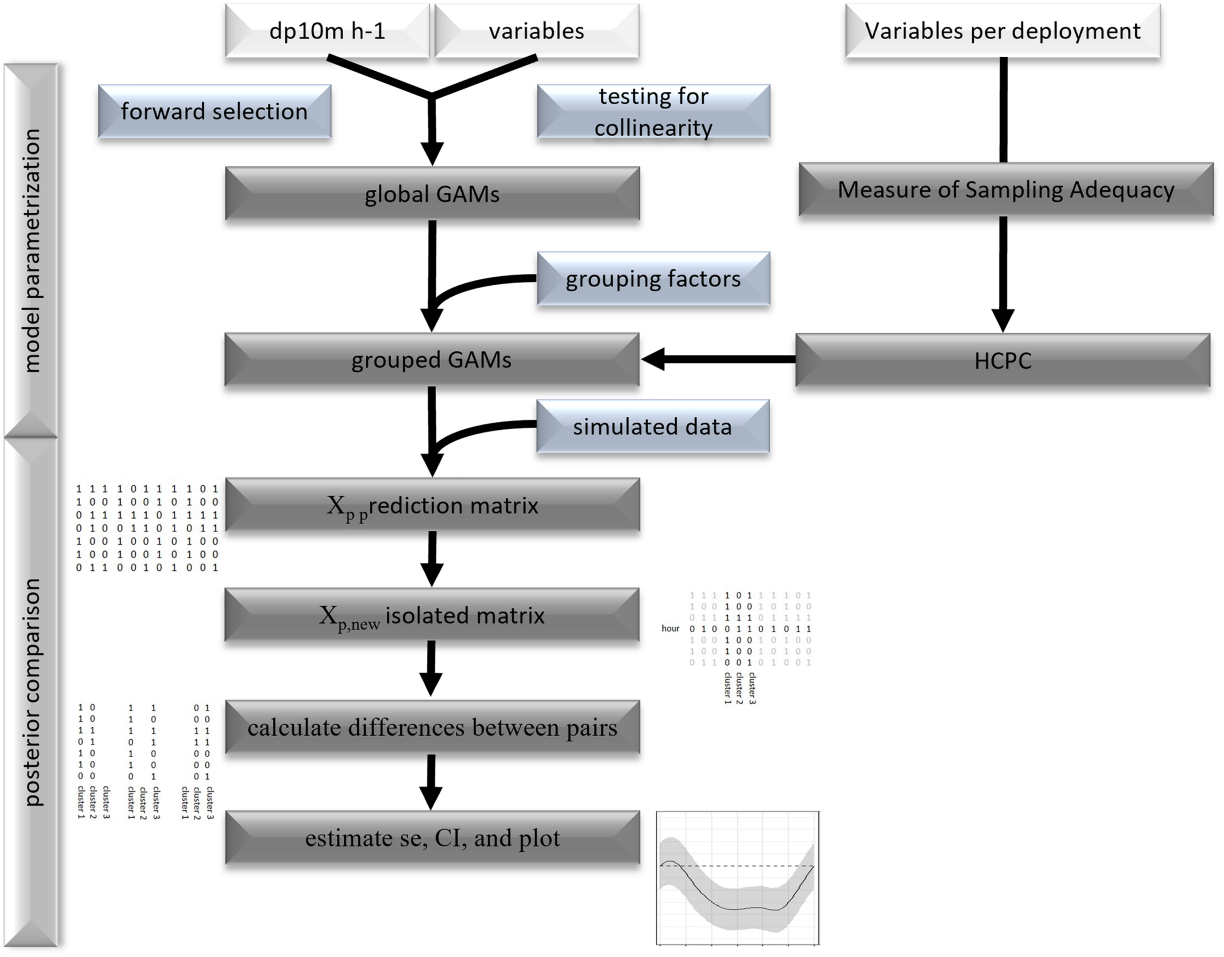
Flowchart of statistical analysis. Flowchart of model parametrization and posterior comparison.

For the posterior comparison of group specific acoustic detections, smooth shapes were generated by exporting model predictions and normalizing one smooth relative to the other (Rose et al., 2012). This was achieved by exporting the prediction matrix (X_p_) that contains the values of the linear predictors (smooth splines) using simulated data. The cluster specific components of the X_p_ matrix were isolated and subtracted from each other (for example: cluster 1-2, 1-3 and 2-3). The resulting matrices X_p(new)_ that were not associated with the smooth terms of the compared pairs were set to 0. Multiplying this matrix with the vector of the fitted coefficients gave the difference between two smooths (here cluster pairs). Then, the standard errors were estimated by multiplying the diagonal elements of X_p(new)_ with the variance covariance matrix ( ${\hat V_\beta }$) of the estimated model coefficients and the transpose of X_p(new)_. From the latter confidence intervals (95%) were generated to identify significant differences between pairs of grouped smooths (Fig. 2, for more details Rose et al., 2012).

Statistical tests, visualization, and modelling were performed with the statistical software R (version 3.6.3, R Core Team, 2020).

## Results

**Figure 3:**
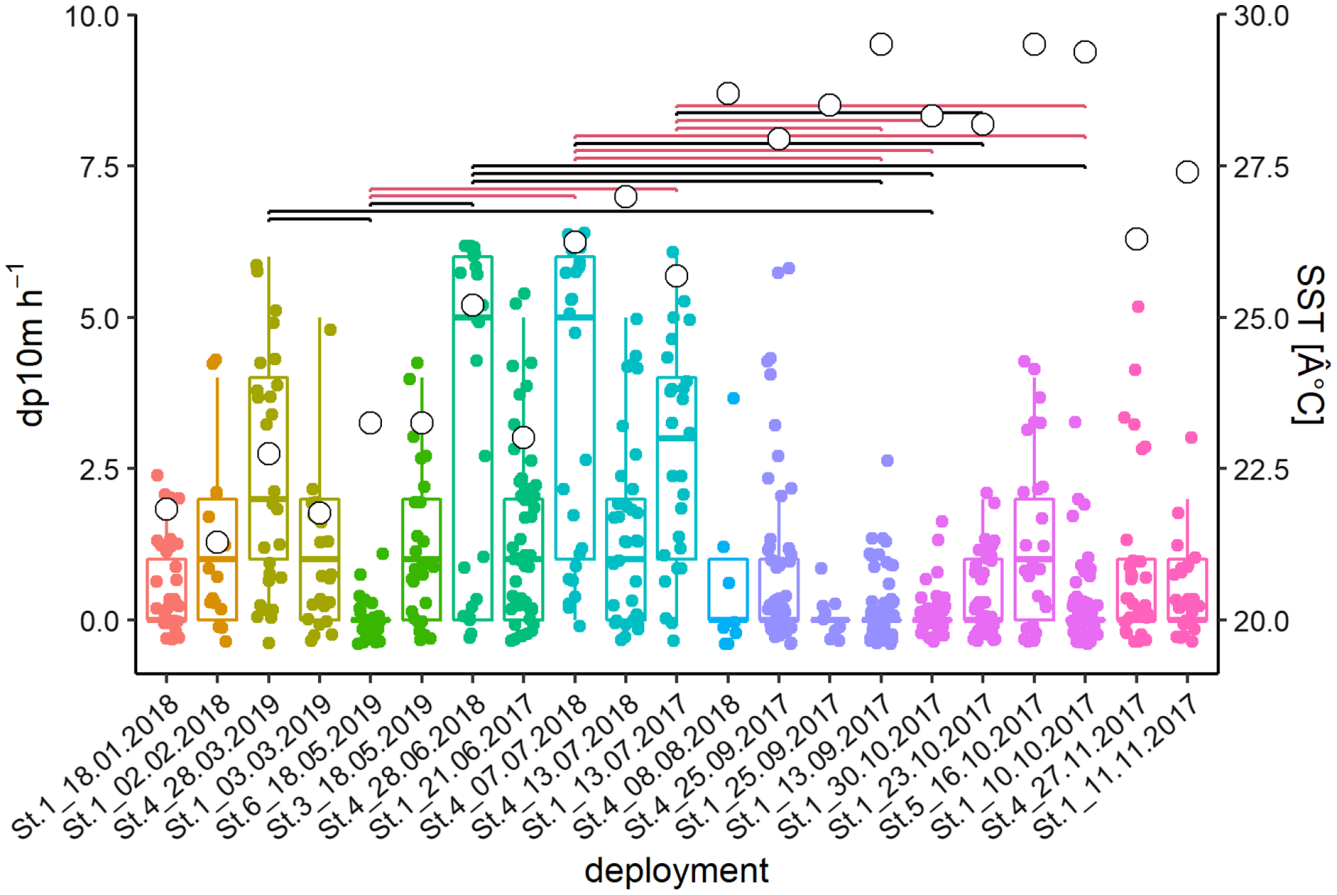
Acoustic data from 21 deployments in the time-frequency domain. Whisker-Boxplot with detection positive 10-min intervals per hour (dp10m h-1) grouped per deployments with Nemenyi test (black < 0.05; red < 0.01) and mean SST (°C, white circles). The representation follows the seasonal regime, but not the chronological order of sampling, please refer to Fig. S1.

The acoustic data from 21 deployments were screened visually in the time-frequency domain. Logged detections (13,442 whistles) were recorded in 266 out of 640 h or 711 out of 3,678 intervals of 10-min, respectively. Kruskal Wallis tests of the latter showed significant differences between deployments and months (deployments: Chi^2^ = 182.2, *p* < 0.001; months: Chi^2^ = 120.44, *p* < 0.001; Fig. 3). Post-hoc tests showed many differences between deployments with high (Jun–Jul) and relatively low (Sep–Oct) detection rates. February and March presented intermediate levels.

**Figure 4:**
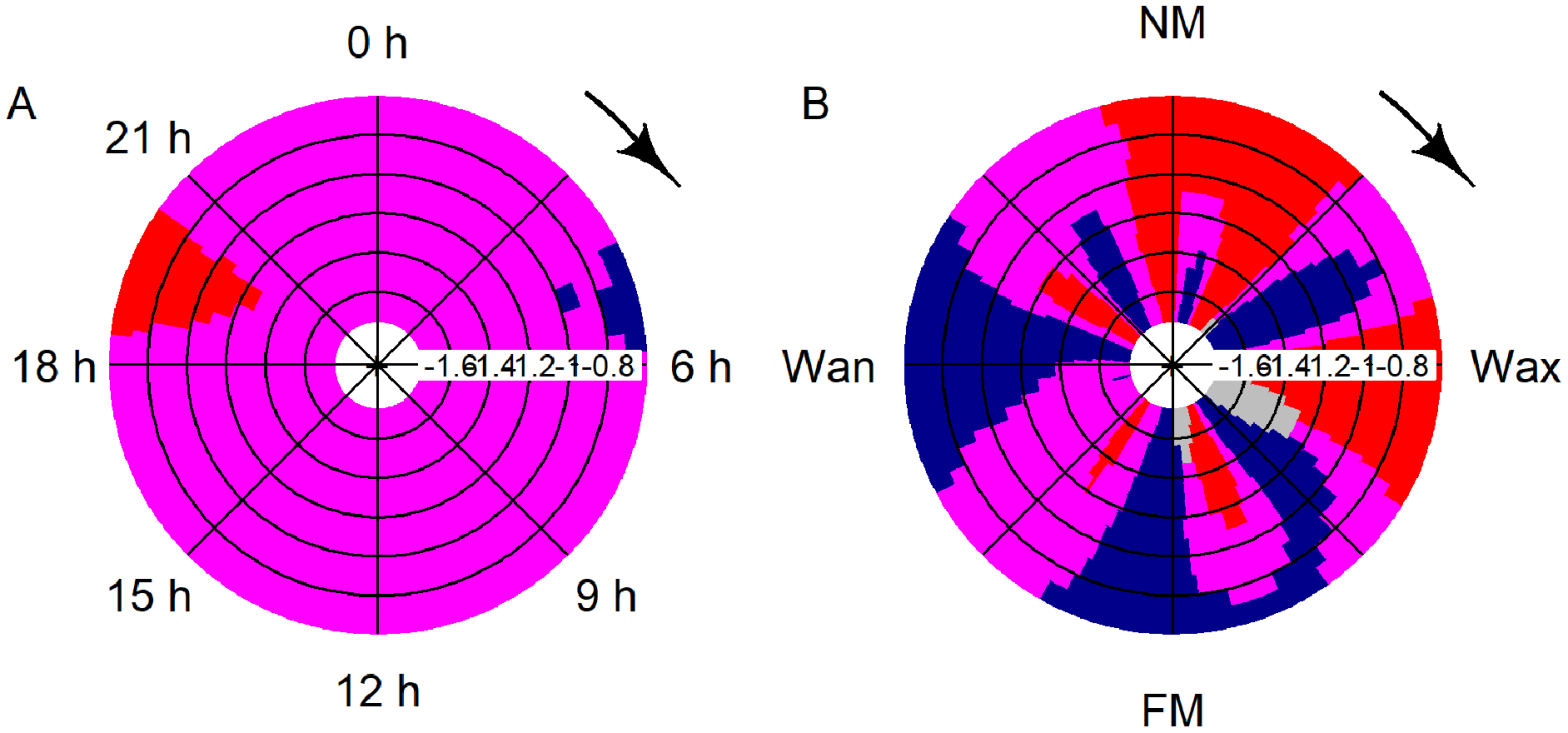
CircSizer map of acoustic data. CircSizer map of acoustic detections relative to hour of the day (A) and moon cycle (B). A blue color pattern indicates a condition, where detections increased significantly, red pattern indicate the opposite, while purple and grey zones refer to areas with no significant changes or data inefficiency, respectively (Oliveira, Crujeiras & Rodríguez-Casal, 2014; NM: new; Wax. waxing; FM: full and Wan: waning moon).

Significant deviation from uniformity was apparent for the variables moon phase and hour (moon: T = 42.1, *p* < 0.001; hour: T = 14.9, *p* < 0.001). Detections decreased significantly between 19:00 and 21:00 and rose between 04:00 and 06:00 (Fig. 4A). Detections were significantly increasing after full, waning and before new moon, while detections were significantly decreasing after new moon and after waxing moon (Fig. 4B).

**Figure 5:**
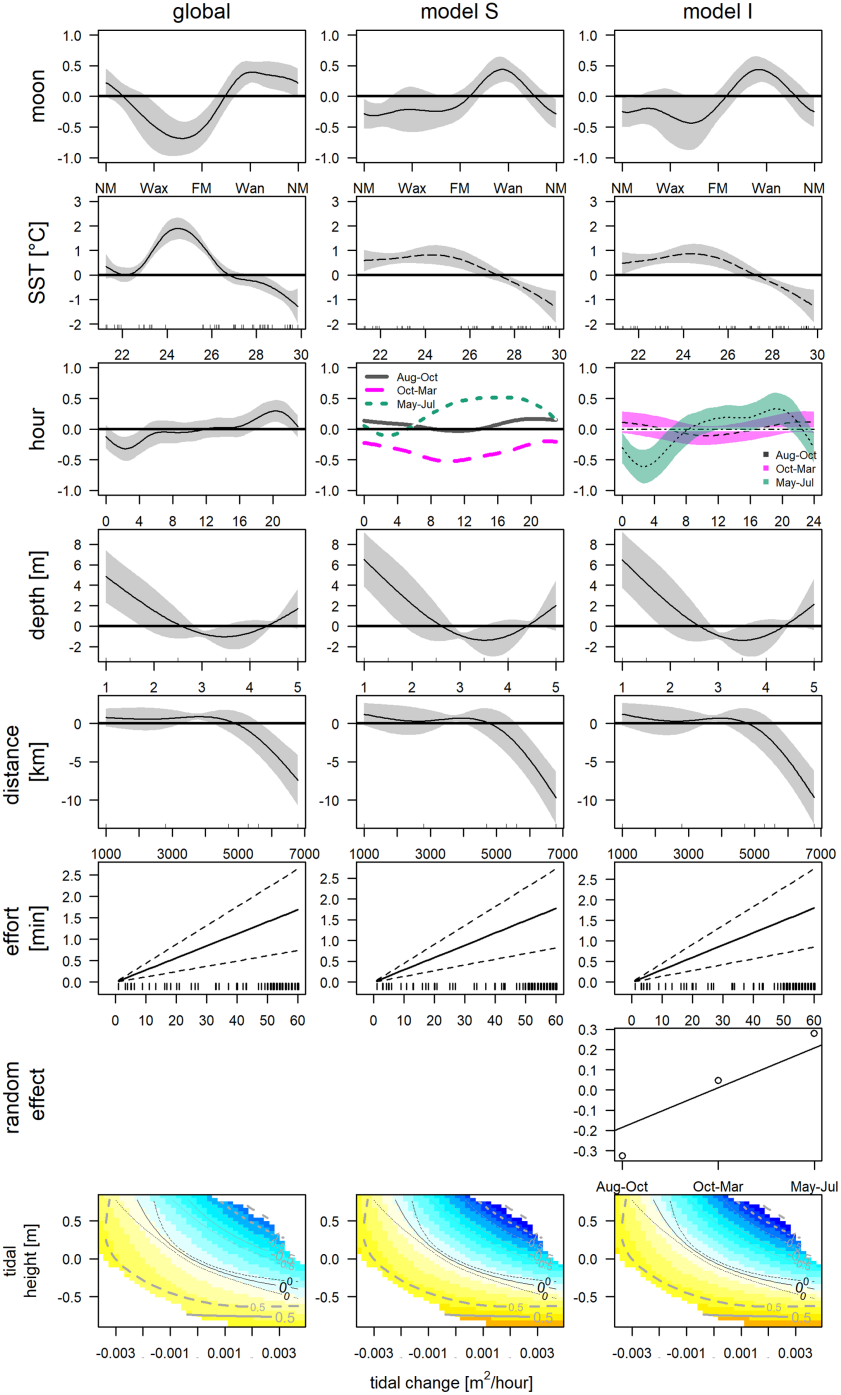
GAMs of acoustic detections. Global and grouped GAMs of acoustic detections in the Ensenada de La Paz. The subplots in the left column visualize the most parsimonious global model (Table 2), in the middle column visualize model S (Table S3), in the right column visualize model I (Table S2). (NM, new moon; Wax, waxing moon; FM, full moon; Wan, waning moon).

The final GAM incorporated nine variables (Table 2, Fig. 5, left column), after excluding variables due to collinearity (Fig. S2) and following forward selection criteria (Table S1, *model 31*). The direct variables accounted for 34.8% (SST: 27.8%, moon phase: 7.3%, tidal condition: 2.6%), the indirect variables for 37.7% (hour: 23.1%, depth: 4.7% and distance: 7.0%) and effort contributed 3.4% of the deviance. This model indicates significantly higher detections between 22.7 °C and 26.4 °C (May, June, Jul and one deployment in November), and significantly lower at temperatures exceeding 27.4 °C (Aug–end of Nov). Furthermore, detections were significantly higher between waning and new moon and significant lower between the waxing and full moon. Bottlenose dolphins were detected during all tidal conditions. However, detections were lower, when water was rising, and the tidal height was relatively high (>−0.26 m). Concerning direct variables: detections were significantly higher between 4:00 and 6:00 and significantly lower between 18:00 and 21:00. Distance indicated that with increasing distance from the entrance of the lagoon detections declined. Additionally, presence was higher if depth was lower than 1.5 m. However, the more distant station was the shallower. The low detection (2 whistles in 24 h) affects the smooths of depth and distance.

**Table 2:** Global generalized additive model of acoustic detection.

Variable	Variable type	Estimate	*p*	Deviance explained	Rel. deviance
All	Intercept	−1.936	3.85e−5	0.385	
s(moon phase)^i^	Cubic cyclic	2.906	<2e−16	0.357	7.3%
te(derivate_tide, tide)^i^	Tensor spline	3.313	9.73e^−03^	0.375	2.6%
s(SST)^i^	Thin plate	6.674	<2e^−16^	0.278	27.8%
s(hour)^d^	Cubic cyclic	4.499	1.03e^−03^	0.296	23.1%
s(depth)^d^	Thin plate	2.13	1.49e^−04^	0.367	4.7%
s(distance)^d^	Thin plate	2.761	1.06e^−05^	0.358	7.0%
Effort^t^	Linear	0.028	3.92e^−04^	0.372	3.4%

**Note:**

Global generalized additive model of acoustic detections in the Ensenada de La Paz. The relative deviance of variables was obtained by obtaining explained deviance when this variable was excluded and keeping coefficients of final model (AIC 1687.01; *N* = 640; see as well Table S1). d, direct, i, indirect, t, technical variables, s, smooth spline; te, tensor spline.

**Figure 6:**
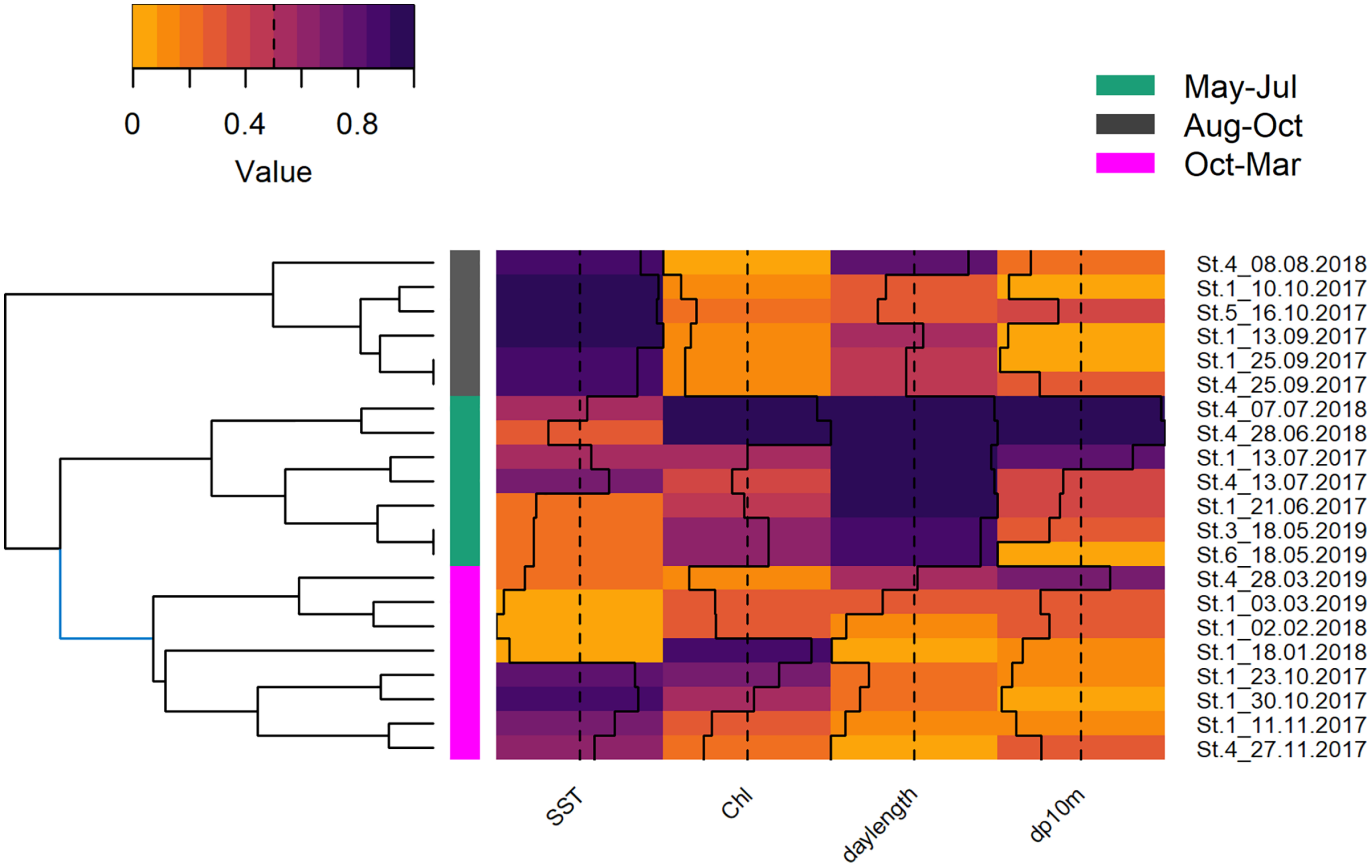
Hierarchical dendrogram of deployments and heatmap with normalized environmental variables. Hierarchical dendrogram of 21 deployments and heatmap with environmental variables used in HCPC and acoustic detections. Variables were normalized before visualization as heatmap (Warnes et al., 2020, see Fig. S3 for more details of HCPC).

The deployments were sampled randomly, hence, it was decided to cluster them by environmental variables instead of using arbitrary periods (**e.g*.*, season, monsoon, Fig. S3). The underlying variables of the HCPC were the first two principal components (82.4%) that resulted from the PCA of the environmental variables that were selected out of a group of seven (SST, Chl and daylength; KMO = 0.52). The obtained clusters separated seven deployments from May, June, and July (May–Jul, 219 h; 406 dp10m), six deployments from August, September, and mid of October (Aug–Oct, 194 h; 121 dp10m) and eight deployments from mid of October until end of March (Oct–Mar, 227 h; 184 dp10m), respectively (Fig. S1). May-Jul was categorized by intermediate SST, intermediate to high chlorophyll and long daylength, Aug–Oct was characterized by high SST, low chlorophyll and intermediate to long daylength, while Oct-Mar was represented by low or high SST, intermediate to high chlorophyll and short to intermediate daylength (Fig. 6). The acoustic detections of these clusters differed significantly (Chi^2^ = 55.534; *p* < 0.001), especially between May–Jul and the other two clusters (Nemenyi test: *p* < 0.001), but not between Aug–Oct and Oct–Mar (Nemenyi test: *p* = 0.28).

The obtained clusters were used as grouping factor of variables SST, moon phase, hour and the tensor spline of tidal height, and derivate flow (16 models, see Table 3). Six out of 16 grouped GAMs had a significant lower AIC score (δAIC < −2, cumulative AIC weight 0.987) and a significant lower deviance than the global GAM. Four of these models were not considered furthermore as they contained one or more insignificant variables that were not grouped and excluding those variables would have provided incomparable model results. The other two models, model S and I (Tables S2 and S3) that grouped the variable hour, retained all variables or all variables but the individual smooths of Aug–Oct and Oct–Mar, respectively (Table 3). Despite a lower AIC score, model I did not differ significantly from model S (δAIC = −6.8). Model S would be selected normally over model I, as model S contained less coefficients, and model I contained two insignificant smooths (Aug–Oct and Oct–Mar), however the AIC weight suggests model I over S (0.351 *vs*. 0.011). These latter results are similar to the univariate Hermans-Rasson test (May–Jul: T = 15.8, *p* < 0.001; Aug–Oct: T = 1.3, *p* > 0.05; Oct–Mar: T = 6.2, *p >* 0.05) indicating that differences were not random. Moreover, model S lacks confidence limits and differently from model I, it is therefore not clear, which smooths deviated really from the mean and how they deviated from each other.

**Table 3:** Grouped generalized additive model of acoustic detection.

Variable	Statistic	GS	S	GI	I
Hour	δAIC	−3.434	−3.396	−9.944	−10.286
	AIC weight	0.011	0.011	0.295	0.35
	Non-statistically significant	Gs-hour	–	Gs-hour; is-hour: Aug-Oct & Oct-Mar	Is-hour: Aug-Oct & Oct-Mar
SST	δAIC	0.152	−0.787	−5.267	−1.169
	AIC weight	0.002	0.003	0.029	0.004
	Non-statistically significant	Tide	Tide	Is-SST: Aug-Oct & Oct-Mar; tide; hour; re: cluster	
Moon	δAIC	1.162	9.467	−9.815	0.15
	AIC weight	0.001	0	0.277	0.002
	Non-statistically significant	Tide	Tide	Is-moon: Aug-Oct & Oct-Mar; tide	Is-moon: Aug-Oct & Oct-Mar; tide
Tide	δAIC	−0.408	2.754	−2.387	−1.062
	AIC weight	0.003	0.001	0.007	0.003
	Non-statistically significant	Gs-tide	Hour	Gs & is-tide; hour	Is-tide hour

**Note:**

Grouped generalized additive model of acoustic detections in the Ensenada de La Paz. δAIC; delta Akaike Information Criterion; gs, global smooth; ss, shared smooth; is, individual smooth; re, random factor; non-statistically significant lists the variables that resulted non-significant (*p* > 0.01).

This uncertainty required a posterior comparison to contrast group specific smooth patterns per model and to compare the resulting patterns among models. These comparisons showed that in both models differences during daytime were present only between Oct-Mar and May-Jul with significantly higher daytime detections in May–Jul in comparison to those in Oct–Mar (model S: 06:00−21:00; model I: 07:00–21:00; Fig. 7). Moreover, common distinctions between the global and these two grouped GAMs consisted in differences in the smooth of moon phase and SST (Fig. 5). The strongest negative effect of the moon phase on the detections of bottlenose dolphins was less expressed in both grouped models than in the global model. Additionally, it was rather apparent during new moon than during waxing and full moon. Moreover, the strongest positive effect of the moon phase in both grouped models was present only during the waning moon. Grouping additional variables both for model S and I tended to overfit the data and did not result in a more meaningful model.

**Figure 7:**
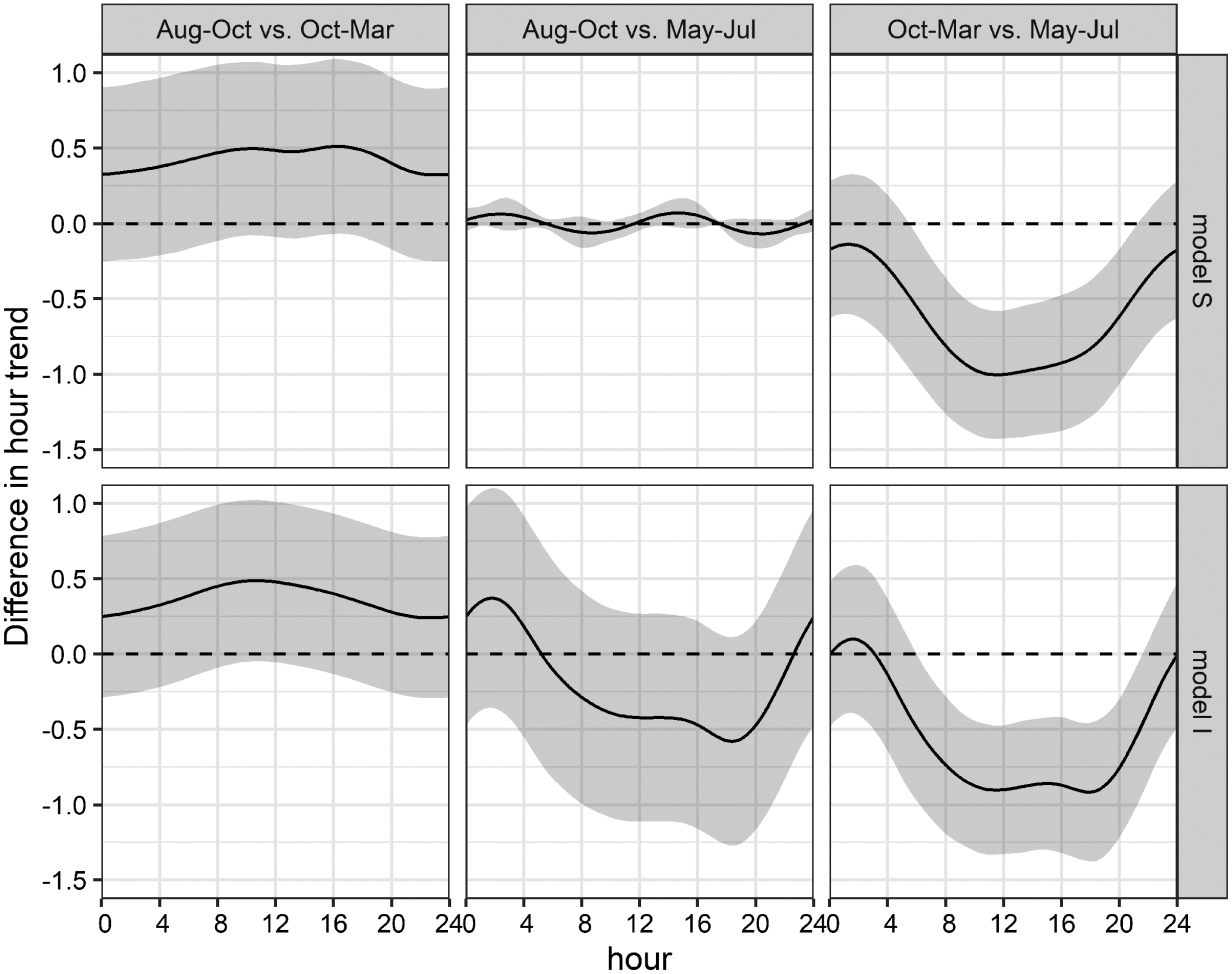
Seasonal differences of daily acoustic activity patterns. Seasonal differences of simulated acoustic detections of bottlenose dolphins in the Ensenada de La Paz comparing each period in both grouped models S and I (Aug–Oct *vs*. Oct–Mar; Aug–Oct *vs*. May–Jul; Oct–Mar *vs*. May–Jul).

## Discussion

Acoustic detections of bottlenose dolphins’ whistles in the Ensenada de La Paz, Baja California Sur, Mexico, varied rhythmically under different cyclic long- and short-term environmental variables as moon phase, tides, and hour. They were highest in March, June, and July, while they were low in May and in August through February. These findings were mostly in line with count data from visual studies conducted in this and adjacent areas over the last thirty to forty years (Acevedo, 1991b; Flores-Ramírez et al., 1996; Salinas Zacarías, 2005; Salvadeo et al., 2009; Marcín-Medina, 2010). Moreover, circular statistics and generalized additive models showed significant deviation from uniformity indicating that bottlenose dolphins’ presence was significantly higher between 04:00 and 6:00 and significantly lower between 18:00 and 21:00. However, differences during the day fluctuated seasonally but only between Oct–Mar and May–Jul with significantly higher daytime detections in May–Jul in comparison to those in Oct–Mar. The available dataset was relatively small but seasonal, moon, tidal, and hourly conditions contained representative values that allowed a generalized analytical approach.

Direct and indirect variables explained the greatest proportion of data deviation of the global GAM. This separation helped to interpret the ecological connections between bottlenose dolphins and their habitat (Guisan & Zimmermann, 2000). For instance, SST may affect bottlenose dolphins directly or indirectly (Wells & Scott, 2002), however, the study area is in the optimal temperature range for bottlenose dolphins, hence SST was considered here only as indirect variable. At temperatures exceeding 27.4 °C (Aug–End of Nov.) bottlenose dolphins occurred less frequently than at temperatures between 22.7 °C and 26.3 °C (May–Jul). Lower detections of bottlenose dolphins in August, when the temperature exceeds 28.7 C, might be a consequence of decreased prey availability (Meekan et al., 2003; Drinkwater et al., 2010). This is the moment when nutrients in the lagoon may start to get depleted (Esqueda-Escárcega et al., 2013) and the abundance of fish and marine mammals is known to decrease, too (González-Acosta et al., 2005; Cubero-Pardo, 2007). Temperature was the only variable related to seasonal productivity patterns, because Chl was excluded due to collinearity (Zuur, Ieno & Elphick, 2010; Dormann et al., 2013). Similarly to SST, Chl is not affecting marine mammals directly, but it is often considered in models as a productivity index of the ecosystem (Redfern et al., 2006; Salvadeo et al., 2009; Pardo et al., 2013; Lopes, 2017). Another indirect variable was the moon phase. The increase in detections during waning and new moon that were observed in this study might be related to increased feeding opportunities, as discussed in other studies (Simonis et al., 2017; Fernandez-Betelu et al., 2019; Cárdenas Hinojosa et al., 2020). Fish are known to respond to changing tidal conditions, which are modulated by the moon phase (Gibson et al., 1996; Gibson, 2003; Gómez-Valdés, Delgado & Dworak, 2003). Hence, while seasonal changing SST may have influenced prey abundance, cyclic patterns such as phases of the moon and tides, both indirect variables, affect their accessibility in terms of changing water depth and hence the overlap of predators and fish assemblages (Hammerschlag, Morgan & Serafy, 2010; Ramos et al., 2011). Very little information exists on how cyclic pattern possibly influence the prey of bottlenose dolphins in the Ensenada de La Paz (Leija-Tristán, de León-González & Rodríguez-Garza, 1992). Also, more studies are necessary to understand the reasons for the rhythmical pattern observed in that study. In a similar way, lower acoustic detections during waxing and full moon might be a result of low prey accessibility, as fish often hide during moonlit nights (Ramos et al., 2011). On the other hand, visual predators see less during the new moon, when their prey venture further away from the protection of mangroves (Ramos et al., 2011), making prey more accessible to those species that do not depend on light, like sharks or dolphins (Hammerschlag, Heithaus & Serafy, 2010; Wang et al., 2015). Moreover, higher tidal levels during new moons can facilitate the access of bigger fish to prey (Ramos et al., 2011), something that most likely is true for marine mammals, too. Nonetheless, moon phase had no significant influence on the nighttime presence of dolphins in the lagoon, which suggests that they were present regardless of the lunar setting.

The acoustic detections in this study indicated that animals were more likely present when the flow of water was entering during flood and high tide. It is possible that they follow fish that are known to migrate in or out of estuaries and lagoons (Shane et al., 1982; Gibson, 2003), while they aggregate along plumes, and in areas with high turbulence, where plankton and fish are known to concentrate (Franks, 1992; Mendes et al., 2002; Reis-Filho, Giarrizzo & Barros, 2016; Cox et al., 2017). This is in line with prior studies that reported how bottlenose dolphins entered the lagoon with tidal changes (Acevedo, 1991b; *Marcín-Medina, 1997)*, while foraging near to the shore (Marcín-Medina, 2010; Moreno-Zuñiga, 2013). Concurrently, it is plausible that their occurrence during low water levels is an indication that they take advantage of low tides, too, while they chased trapped animals. These specific techniques are known from the northern Gulf of California (Leatherwood, 1975; Silber & Fertl, 1995; Lewis & Schroeder, 2003). Neverthless, it is normally a coordinated effort of a group of bottlenose dolphins, while animals in the Ensenada de La Paz mostly entered in groups but forage alone (Marcín-Medina, 1997, 2010).

Concerning direct variables, slightly lower detections at night (20:00–04:00) and the maximal peak before dusk as registered in this study might result from animals that forage less at night but more at twilight. This is in line with higher biomass and lower diversity levels at night that are commonly observed in fish assemblages, which are related to predators foraging activity and strategies to avoid them (Zárate-Hernández et al., 2012). However, the differences in acoustic detections were low and this might highlight the continued nature in which bottlenose dolphins frequent the lagoon. This might indicate that food accessibility is relatively constant during day and night, the influence of a less important factor for frequenting this lagoon, or that whistles are not as an ideal index of bottlenose dolphins’ presence as echolocation clicks. The first two aspects are in line with prior investigations that stressed the lagoons potentially critical value not only for feeding but as well for resting, socializing, and nursing bottlenose dolphins (Marcín-Medina, 1997, 2010), because, in these behaviors, whistles tend to be emitted more frequently (Díaz López, 2011; King & Janik, 2015). The third aspect is relevant, too, because whistling might be discontinuous and solitary dolphins tend to whistle less (Oswald, Rankin & Barlow, 2008). In some acoustic studies that analyzed both clicks and whistles, emissions did not modulate synchronously (Baumann-Pickering et al., 2015; Lin et al., 2015; Caruso et al., 2017). Therefore, animals might be present but do not emit whistles during foraging, or might increase whistling as they use it to coordinate their foraging effort (King & Janik, 2015). This aspect is difficult to prove, and it would apply both for day and nighttime. Low frequency components of clicks and feeding buzzes were sporadically recorded in this study, but almost only when whistles were recorded too. The directionality and frequency dependent absorption of echolocation clicks can reduce the probability of detection (Au, 1993; Finneran et al., 2014). Off axis clicks that might be recorded in silent environments might be masked by the noises produced by snapping shrimps and tidal currents. Hence to sample quantitatively, a higher number of hydrophones would have been required to record present animals effectively. Moreover, this study did not focus primarily on foraging strategies or habitats. Here whistles were rather chosen not only for technical and cost related considerations, but as well to highlight the use of the lagoon for socialization, something that recordings of echolocation clicks from bottlenose dolphins cannot provide. In this way the study verified the advantages of custom-made hydrophones that were used effectively and allowed a reasonable economic investment while realizing this research.

The negative relation between the presence bottlenose dolphins and the water depth and distance to the entrance of the lagoon has been shown in other studies (Ingram & Rogan, 2002; Bowen, 2011). However, it needs to be taken into consideration that acoustical methods can detect bottlenose dolphins in large distances, hence the depth might not be taken literally. Five out of six stations were positioned in the middle or the boarder of the channel, while station 6 is situated 1.0 km away from the channel in 1.0 m depth. The low depth and interactions with the muddy sediment may reduce the propagation of sound (Ballard & Lee, 2017). Bottlenose dolphin do not venture very far in this area as has been shown by observational studies (Marcín-Medina, 2010), however their resting areas are preferably in the lagoon in shallow areas. It is furthermore less likely to record clicks or whistles from resting animals (Sekiguchi & Kohshima, 2003). Hence, it is very likely that resting behavior and sound propagation in shallow water with muddy sediment were the main reason for the low at station 6.

The overall hourly vocalization rate in this study was biased due to high rates and a strong pattern throughout the day in May, June, and July. This required to split the analysis by seasons. Seasonally changing diel presence and activity pattern have been reported before for odontocete species (Fernandez-Betelu et al., 2019; Osiecka, Jones & Wahlberg, 2020; Gregorietti et al., 2021). Fernandez-Betelu et al. (2019) discussed how diel patterns of emitted click sounds changed seasonally by studying when monthly circular patterns of tidal and diel terms changed. They concluded that differing activity patterns of bottlenose dolphins might result from changing productivity and prey patterns. Similarly, Gregorietti et al. (2021) concluded that diel whistle patterns that originated from bottlenose dolphins change seasonally. Differently, Osiecka, Jones & Wahlberg (2020) observed that captive harbor porpoises showed strong daily vocalization pattern with higher vocalizations at crepuscular hours. Moreover, during short summer nights, crepuscular peaks appeared as one maximum, whereas two peaks were found during all other seasons. As the animals were fed only during daytime hours (09:00–15:30), vocalizations were unrelated to feeding events. They concluded that diel vocalizations in the wild were not determined only by the diel patterns of their prey but by the change of light availability, too. The latter is an important finding because porpoises use click trains not only for orientation and for foraging but for social interactions, too (Osiecka, Jones & Wahlberg, 2020).

Posterior tests are uncommon in GAMs, however, group specific normalization of two factors at a time has been already previously proposed for GAMs with individual penalties (Rose et al., 2012). Moreover, in GAMs with shared penalties the comparison of grouped smooth in a standard display is complicated by the lack of confidence bands making a paired comparison attractive. That is why model I should be preferred over model S, if a posterior analysis is not possible. This type of comparison might be interesting in cases where data differ substantially among groups. This might indeed increase uncertainties, as GAMs are normalized to the mean and might react more sensible to unbalanced datasets. In this study, data of the tree clusters contained roughly equal numbers (219, 194 and 227 h), but number of detections were 2.2 and 3.4 times higher in May–Jul than in the other two periods. This resulted, together with a relatively similar tidal condition of the May–Jul data, in a sustained effect on the variable hour in all models, what finally might have obscured the relevance that tidal conditions have on this model. A possible approach to circumvent this issue might be the inclusion of day and night as categorical variables instead of hour. Alternative modelling approaches include Generalized Equation Estimation or neural networks that might be more flexible especially as the possibility to include interaction terms among variables are less limited than in GAMs.

## Conclusions

Bottlenose dolphins in the Ensenada de La Paz were detected continuously throughout the year, however their presence fluctuated seasonally and under diverse cyclic patterns. The separation in prey related and dolphin related variables allowed to highlight the ecological space in which bottlenose dolphins exist. Moreover, it showed how variables that influenced them indirectly had a slightly higher influence on the model results than direct variables.

Indirect variables were related to environmental conditions that favor prey concentration and availability as SST, moon, and tidal phases. Concerning direct variables, water depth and distance from the entrance of the lagoon were found to influence bottlenose dolphin presence, related probably to feeding, social, and resting needs. Finally, differences through the day that may have both direct and indirect implications resulted as significant especially when interactions with seasonal conditions were considered. The latter highlights the benefit of grouped over global GAMs when seasonal difference suggest that marine mammals display distinct behavior and require a more flexible analytical approach.

The bottlenose dolphin is considered a sentinel species for environmental changes and its widespread distribution could allow it to obtain valuable information about other coastal areas. This is especially important as these habitats provide resources of high quantity but as well quality. Therefore, conservation management should focus on identifying relevant food sources and limit the actions that have negative effects on their abundance and distribution in the habitat of bottlenose dolphins.

## Supplemental Information

10.7717/peerj.13246/supp-1 Supplemental Information 1 R script to obtain Fig. 3. Acoustic data from 21 deployments in the time-frequency domain. Whisker-Boxplot with detection positive 10-min intervals per hour (dp10m h-1) grouped per deployments with Nemenyi test (black < 0.05; red < 0.01) and mean SST (°C, white circles). The representation follows the seasonal regime, but not the chronological order of sampling, please refer to Fig. S1.

10.7717/peerj.13246/supp-2 Supplemental Information 2 R script to obtain Fig. 4. CircSizer map of acoustic data. CircSizer map of acoustic detections relative to hour of the day (A) and lunar cycle (B). A blue color pattern indicates a condition, where detections increased significantly, red pattern indicate the opposite, while purple and grey zones refer to areas with no significant changes or data inefficiency, respectively (Oliveira, Crujeiras & Rodríguez-Casal, 2014; NM: new; Wax. waxing; FM: full and Wan: waning moon).

10.7717/peerj.13246/supp-3 Supplemental Information 3 CSV file for R script to obtain Fig. 4. Global and grouped GAMs of acoustic detections in the Ensenada de La Paz (NM: new moon, 1. Qu: waxing moon, FM: full moon, 3. Qu: waning moon).

10.7717/peerj.13246/supp-4 Supplemental Information 4 R script to obtain Fig. 5. Hierarchical dendrogram of 21 deployments and heatmap with normalized environmental variables used in HCPC as well as normalized acoustic detections.

10.7717/peerj.13246/supp-5 Supplemental Information 5 R script to obtain Fig. 6. Hierarchical dendrogram of 21 deployments and heatmap with environmental variables used in HCPC and acoustic detections. Variables were normalized before visualization as heatmap (Warnes et al., 2020, see Fig. S4 for more details of HCPC).

10.7717/peerj.13246/supp-6 Supplemental Information 6 R script to obtain Fig. 7. Seasonal differences of simulated acoustic detections of bottlenose dolphins in the Ensenada de La Paz comparing each period in both grouped models S and I (Aug-Oct *vs*. Oct-Mar; Aug-Oct *vs*. May-Jul; Oct-Mar *vs*. May-Jul).

10.7717/peerj.13246/supp-7 Supplemental Information 7 R script to obtain Table 2. Global generalized additive model of acoustic detection in the Ensenada de La Paz (N = 640, see as well Table S1, d: direct, i: indirect variable).

10.7717/peerj.13246/supp-8 Supplemental Information 8 R script to obtain Table 3. Grouped generalized additive model of acoustic detection in the Ensenada de La Paz (gs: global smooth; ss: shared smooth; is: individual smooth; re: random factor).

10.7717/peerj.13246/supp-9 Supplemental Information 9 Detection positive 10 minute intervals during 21 deployments relative to effort in hours, tidal height, moon phase, and day time. Detection positive 10-minute intervals (black points) during 21 deployments relative to effort in hours, tidal height, moon phase and daytime (start date in right upper corner, grey shaded rectangles: night time) per HCPC cluster (left: Aug-Oct; middle: Oct-Mar; right: May-Jul, *: data from Gauger, Caraveo-Patiño & Romero-Vivas 2020).

10.7717/peerj.13246/supp-10 Supplemental Information 10 R script to obtain Fig. S1. Detection positive 10-minute intervals (black points) during 21 deployments relative to effort in hours, tidal height, moon phase and daytime (start date in right upper corner, grey shaded rectangles: night time) per HCPC cluster (left: Aug-Oct; middle: Oct-Mar; right: May-Jul, *: data from Gauger, Caraveo-Patiño & Romero-Vivas 2020).

10.7717/peerj.13246/supp-11 Supplemental Information 11 Correlation plot tested for collinearity of variables considered during model selection. Numbers were printed only if Spearman rank correlation were significant.

10.7717/peerj.13246/supp-12 Supplemental Information 12 R script to obtain Fig. S2. Correlation plot tested for collinearity of variables considered during model selection (numbers were printed only if Spearman rank correlation were significant).

10.7717/peerj.13246/supp-13 Supplemental Information 13 Cluster analysis of 21 deployments according to the two principal components of environmental data. Hierarchical dendrogram (A) and hierarchical clustering (B) of 21 deployments according to the two principal components of environmental data.

10.7717/peerj.13246/supp-14 Supplemental Information 14 R script to obtain Fig. S3. Hierarchical dendrogram (A) and hierarchical clustering (B) of 21 deployments according to the two principal components of environmental data.

10.7717/peerj.13246/supp-15 Supplemental Information 15 Models, following forward selection criteria. Forward variable selection of detection positive 10-minutes with bottlenose dolphin whistles per hour as a function of environmental variables.

10.7717/peerj.13246/supp-16 Supplemental Information 16 R script to obtain Table S1. Forward variable selection of detection positive 10-minutes with bottlenose dolphin whistles per hour as a function of environmental variables.

10.7717/peerj.13246/supp-17 Supplemental Information 17 CSV file for R script to obtain Fig. S1. Dataset with detection positive 10-minute intervals per hour of 21 deployments.

10.7717/peerj.13246/supp-18 Supplemental Information 18 The coefficients of model I. The coefficients of model I, a GAM that deviates from the global GAM by the variable hour. Its penalties were granted individually per cluster.

10.7717/peerj.13246/supp-19 Supplemental Information 19 R script to obtain supplemental table_S2. The coefficients of model I, a GAM that deviates from the global GAM by the variable hour. Its penalties were granted individually per cluster.

10.7717/peerj.13246/supp-20 Supplemental Information 20 CSV file for R script to obtain Table S2. The coefficients of model I, a GAM that deviates from the global GAM by the variable hour. Its penalties were granted individually per cluster.

10.7717/peerj.13246/supp-21 Supplemental Information 21 The coefficients of model S. The coefficients of model S, a GAM that deviates from the global GAM by the variable hour. Its penalties were shared between all three clusters.

10.7717/peerj.13246/supp-22 Supplemental Information 22 R script to obtain Table S3. The coefficients of model S, a GAM that deviates from the global GAM by the variable hour. Its penalties were shared between all three clusters.

## Additional Information and Declarations

### Data Availability

The following information was supplied regarding data availability: The raw measurements are available in the Supplemental Files. Statistical tests, visualization, and modeling were performed with the statistical software R (version 3.6.3, R Core Team, 2020).
