# Supplementary figures and images for: Seasonal and diel influences on bottlenose dolphin acoustic detection determined by whistles in a coastal lagoon in the southwestern Gulf of California
Source: PeerJ. 2022 May 18;10:e13246. doi: 10.7717/peerj.13246 (PMC9123887; doi:10.7717/peerj.13246)

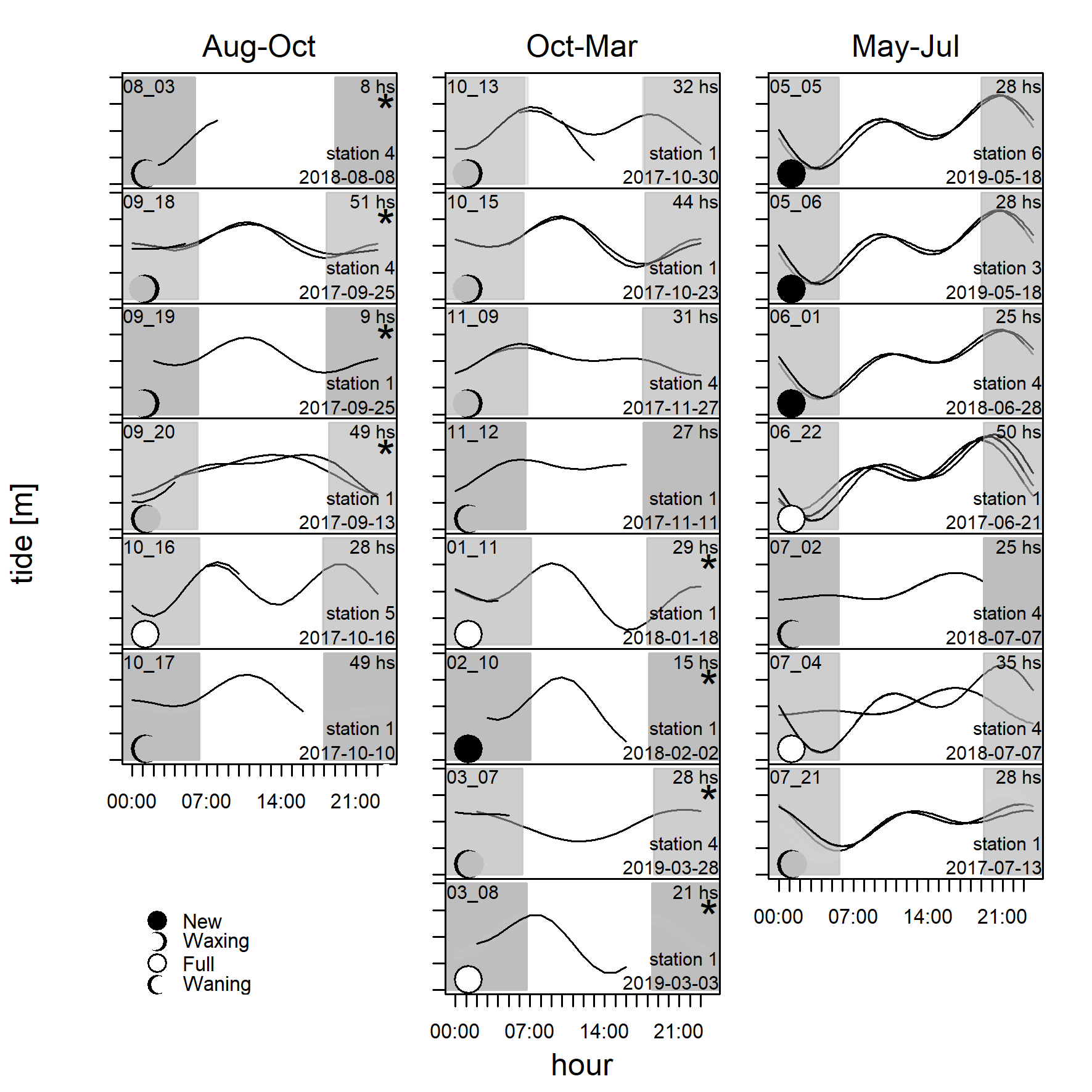

Supplement: Supplemental Information 9 — Detection positive 10-minute intervals (black points) during 21 deployments relative to effort in hours, tidal height, moon phase and daytime (start date in right upper corner, grey shaded rectangles: night time) per HCPC cluster (left: Aug-Oct; middle: Oct-Mar; right: May-Jul, *: data from Gauger, Caraveo-Patiño & Romero-Vivas 2020). [file peerj-10-13246-s009.png]

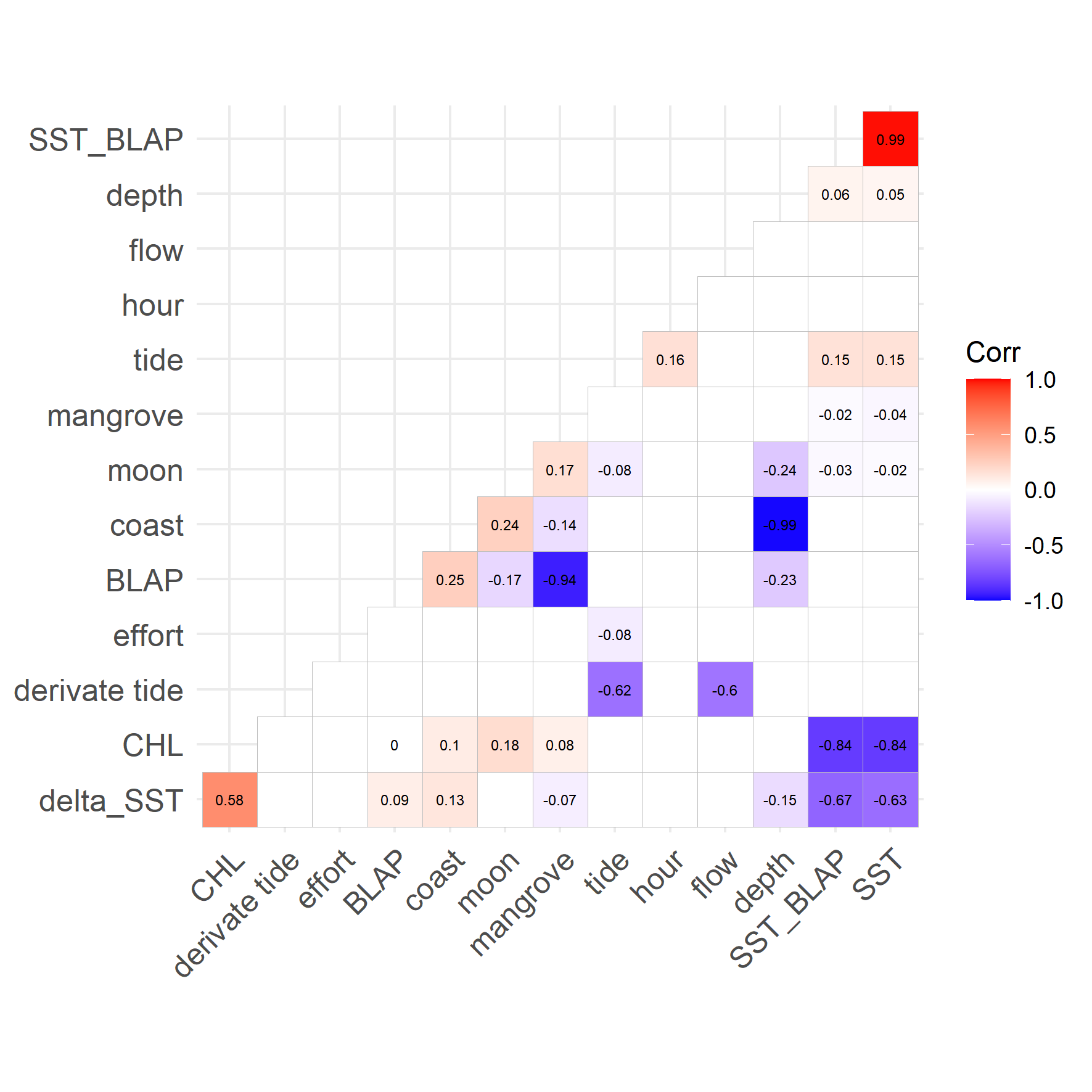

Supplement: Supplemental Information 11 — Numbers were printed only if Spearman rank correlation were significant. [file peerj-10-13246-s011.png]

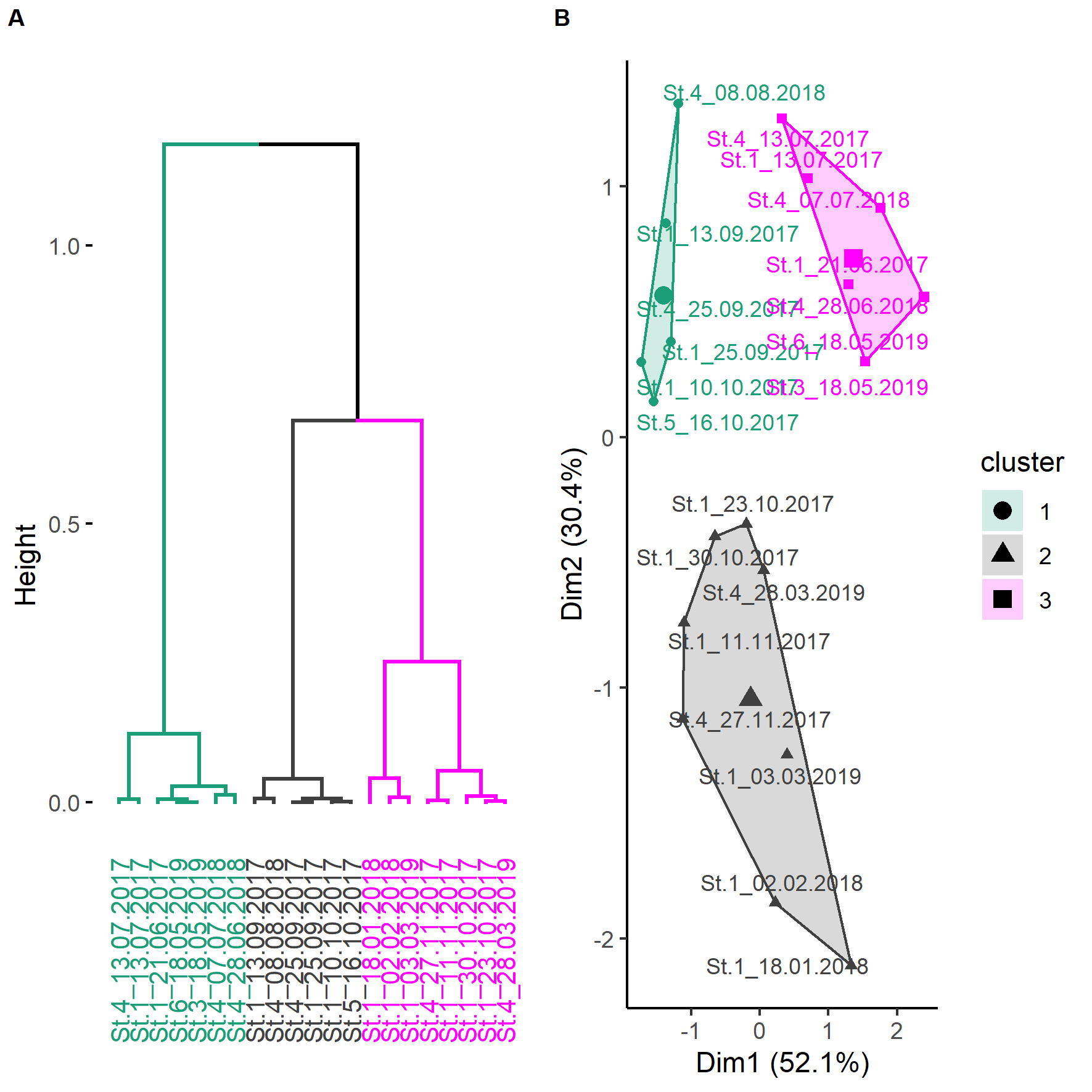

Supplement: Supplemental Information 13 — Hierarchical dendrogram (A) and hierarchical clustering (B) of 21 deployments according to the two principal components of environmental data. [file peerj-10-13246-s013.png]
