# Supplementary material for: Seasonal and diel influences on bottlenose dolphin acoustic detection determined by whistles in a coastal lagoon in the southwestern Gulf of California
Source: PeerJ. 2022 May 18;10:e13246. doi: 10.7717/peerj.13246 (PMC9123887; doi:10.7717/peerj.13246)
Supplement: Supplemental Information 15 — Forward variable selection of detection positive 10-minutes with bottlenose dolphin whistles per hour as a function of environmental variables. [file peerj-10-13246-s015.docx]

Table S1:

Forward variable selection of detection positive 10-minutes with bottlenose dolphin whistles per hour as a function of environmental variables.

dp10m h^-1^: detection positive 10-minute intervals with bottlenose dolphin whistles per hour; s: smooth spline; te: tensor spline; cc: cubic cyclic spline; * represents global model

| model | formula | AIC | dAIC | df | AIC weight | **comment** |
| --- | --- | --- | --- | --- | --- | --- |
| 01 | dp10m_ELAP1 ~ 1 | 2173.58 | 488.55 | 1.00 | 0.000 |  |
| 02 | dp10m_ELAP1 ~ s(SST) | 1906.80 | 221.77 | 8.36 | 0.000 |  |
| 03 | dp10m_ELAP1 ~ s(SST_BLAP) | 1908.41 | 223.38 | 7.71 | 0.000 |  |
| 04 | dp10m_ELAP1 ~ s(delta_SST) | 2052.67 | 367.64 | 8.92 | 0.000 |  |
| 05 | dp10m_ELAP1 ~ s(CHL_BLAP) | 1975.70 | 290.66 | 9.82 | 0.000 |  |
| 06 | dp10m_ELAP1 ~ s(lunar_phase, bs = "cc", k = 6) | 2120.92 | 435.89 | 4.71 | 0.000 |  |
| 07 | dp10m_ELAP1 ~ s(flow) | 2156.32 | 471.29 | 8.22 | 0.000 |  |
| 08 | dp10m_ELAP1 ~ s(derivate_tide) | 2174.70 | 489.67 | 2.00 | 0.000 |  |
| 09 | dp10m_ELAP1 ~ s(tide) | 2145.36 | 460.33 | 9.35 | 0.000 |  |
| 10 | dp10m_ELAP1 ~ effort | 2145.23 | 460.20 | 2.00 | 0.000 |  |
| 11 | dp10m_ELAP1 ~ s(h, bs = "cc", k = 10) | 2173.60 | 488.56 | 1.01 | 0.000 |  |
| 12 | dp10m_ELAP1 ~ s(depth, k = 4) | 1960.80 | 275.77 | 4.00 | 0.000 |  |
| 13 | dp10m_ELAP1 ~ s(distance_BLAP, k = 4) | 1956.04 | 271.00 | 4.00 | 0.000 |  |
| 14 | dp10m_ELAP1 ~ s(distance_coast, k = 4) | 2056.07 | 371.03 | 3.86 | 0.000 |  |
| 15 | dp10m_ELAP1 ~ s(distance_mangrove, k = 4) | 1966.40 | 281.37 | 4.00 | 0.000 |  |
| 16 | dp10m_ELAP1 ~ s(SST) + s(lunar_phase, bs = "cc", k = 6) | 1843.43 | 158.40 | 12.66 | 0.000 |  |
| 17 | dp10m_ELAP1 ~ s(SST) + s(tide) | 1909.05 | 224.01 | 10.88 | 0.000 |  |
| 18 | dp10m_ELAP1 ~ s(SST) + s(lunar_phase, bs = "cc", k = 6) + s(tide) | 1845.16 | 160.13 | 13.66 | 0.000 |  |
| 19 | dp10m_ELAP1 ~ s(SST) + s(lunar_phase, bs = "cc", k = 6) + s(flow) | 1843.89 | 158.86 | 16.49 | 0.000 |  |
| 20 | dp10m_ELAP1 ~ s(SST) + s(lunar_phase, bs = "cc", k = 6) + s(derivate_tide) | 1843.93 | 158.89 | 14.12 | 0.000 |  |
| 21 | dp10m_ELAP1 ~ s(SST) + s(lunar_phase, bs = "cc", k = 6) + te(flow) | 1843.37 | 158.33 | 15.98 | 0.000 |  |
| 22 | c("dp10m_ELAP1 ~ s(SST) + s(lunar_phase, bs = "cc", k = 6) + te(flow, tide, bs = c("tp", "tp"), k = c(10, 10))") | 1844.01 | 158.97 | 20.94 | 0.000 |  |
| 23 | c("dp10m_ELAP1 ~ s(SST) + s(lunar_phase, bs = "cc", k = 6) + te(derivate_tide, tide, bs = c("tp", "tp"), k = c(10, 10))") | 1830.41 | 145.38 | 48.97 | 0.000 |  |
| 24 | c("dp10m_ELAP1 ~ s(lunar_phase, bs = "cc", k = 6) + te(flow, tide, bs = c("tp", "tp"), k = c(10, 10)) + s(SST, k = 10, bs = "tp", m = 2)") | 1844.01 | 158.97 | 20.94 | 0.000 |  |
| 25 | c("dp10m_ELAP1 ~ s(SST) + s(lunar_phase, bs = "cc", k = 6) + s(derivate_tide) + s(h, bs = "cc", k = 6)") | 1844.63 | 159.59 | 15.85 | 0.000 |  |
| 26 | c("dp10m_ELAP1 ~ s(SST) + s(lunar_phase, bs = "cc", k = 6) + s(derivate_tide) + s(tide) + s(h, bs = "cc", k = 6)") | 1838.23 | 153.20 | 17.85 | 0.000 |  |
| 27 | c("dp10m_ELAP1 ~ s(SST) + s(lunar_phase, bs = "cc", k = 6) + te(derivate_tide, tide, bs = c("tp", "tp"), k = c(10, 10)) + effort") | 1818.07 | 133.03 | 49.35 | 0.000 |  |
| 28 | c("dp10m_ELAP1 ~ s(SST, k = 10, bs = "tp", m = 2) + s(lunar_phase, bs = "cc", k = 6) + te(derivate_tide, tide, bs = c("tp", "tp"), k = c(10, 10)) + s(h, bs = "cc") + effort") | 1813.91 | 128.88 | 22.29 | 0.000 |  |
| 29 | c("dp10m_ELAP1 ~ s(lunar_phase, bs = "cc", k = 6) + te(derivate_tide, tide, bs = c("tp", "tp"), k = c(10, 10)) + s(SST, k = 10, bs = "tp", m = 2) + s(h, bs = "cc") + effort + s(depth, k = 4)") | 1721.88 | 36.84 | 26.37 | 0.000 |  |
| 30 | c("dp10m_ELAP1 ~ s(lunar_phase, bs = "cc", k = 6) + te(derivate_tide, tide, bs = c("tp", "tp"), k = c(10, 10)) + s(SST, k = 10, bs = "tp", m = 2) + s(h, bs = "cc") + effort + s(depth, k = 4) + s(delta_SST)") | 1720.97 | 35.93 | 28.34 | 0.000 |  |
| 31 | c("dp10m_ELAP1 ~ s(lunar_phase, bs = "cc", k = 6) + te(derivate_tide, tide, bs = c("tp", "tp"), k = c(10, 10)) + s(SST, k = 10, bs = "tp", m = 2) + s(h, bs = "cc") + effort + s(depth, k = 4) + s(distance_BLAP, k = 4)") | 1687.01 | 1.98 | 27.31 | 0.218 | ***;** most parsimonious model |
| 32 | c("dp10m_ELAP1 ~ s(lunar_phase, bs = "cc", k = 6) + te(derivate_tide, tide, bs = c("tp", "tp"), k = c(10, 10)) + s(SST, k = 10, bs = "tp", m = 2) + s(h, bs = "cc") + effort + s(depth, k = 4) + s(distance_BLAP, k = 4) + s(distance_coast, k = 5)") | 1687.24 | 2.20 | 26.83 | 0.195 | depth and distance_mangrove not significant |
| 33 | c("dp10m_ELAP1 ~ s(lunar_phase, bs = "cc", k = 6) + te(derivate_tide, tide, bs = c("tp", "tp"), k = c(10, 10)) + s(SST, k = 10, bs = "tp", m = 2) + s(h, bs = "cc") + effort + s(depth, k = 4) + s(distance_BLAP, k = 4) + s(distance_coast, k = 5) + s(distance_mangrove, k = 5)") | 1685.03 | 0.00 | 26.22 | 0.587 | **delta_SST not significant** |
