# Supplementary material for: Seasonal and diel influences on bottlenose dolphin acoustic detection determined by whistles in a coastal lagoon in the southwestern Gulf of California
Source: PeerJ. 2022 May 18;10:e13246. doi: 10.7717/peerj.13246 (PMC9123887; doi:10.7717/peerj.13246)
Supplement: Supplemental Information 18 — The coefficients of model I, a GAM that deviates from the global GAM by the variable hour. Its penalties were granted individually per cluster. [file peerj-10-13246-s018.docx]

Table S2:

The coefficients of model I, a GAM that deviates from the global GAM by the variable hour. Its penalties were granted individually per cluster.

| coefficients | estimation | error est. | t | p | significance |
| --- | --- | --- | --- | --- | --- |
| (intercept) | -2.06 | 0.51 | -4.00 | 0.000 | *** |
| effort | 0.03 | 0.01 | 3.78 | 0.000 | *** |
| smooths | edf | Redf | Chi² | p | significance |
| s(moon phase) | 3.43 | 8.00 | 35.90 | 0.000 | *** |
| te(derivate tide, tide) | 3.00 | 3.00 | 17.20 | 0.001 | *** |
| s(SST) | 3.47 | 4.18 | 43.60 | 0.000 | *** |
| s(hour): cluster Aug-Oct | 0.00 | 8.00 | 0.00 | 0.738 |  |
| s(hour): cluster Oct-Mar | 1.18 | 8.00 | 2.38 | 0.114 |  |
| s(hour): cluster May-Jul | 4.03 | 8.00 | 23.80 | 0.000 | *** |
| s(depth) | 2.14 | 2.17 | 31.60 | 0.000 | *** |
| s(distance) | 2.79 | 2.82 | 44.00 | 0.000 | *** |
| s(cluster) | 1.45 | 2.00 | 15.00 | 0.000 | *** |
