# Supplementary material for: Seasonal and diel influences on bottlenose dolphin acoustic detection determined by whistles in a coastal lagoon in the southwestern Gulf of California
Source: PeerJ. 2022 May 18;10:e13246. doi: 10.7717/peerj.13246 (PMC9123887; doi:10.7717/peerj.13246)
Supplement: Supplemental Information 21 — The coefficients of model S, a GAM that deviates from the global GAM by the variable hour. Its penalties were shared between all three clusters. [file peerj-10-13246-s021.docx]

Table S3:

The coefficients of model S, a GAM that deviates from the global GAM by the variable hour. Its penalties were shared between all three clusters.

| Coefficients | estimation | error est. | t | p | significance |
| --- | --- | --- | --- | --- | --- |
| (intercept) | -2.03 | 0.52 | -3.92 | 0.0001 | *** |
| effort | 0.03 | 0.01 | 3.74 | 0.0002 | *** |
| smooths | edf | Redf | Chi² | p | significance |
| s(moon phase) | 2.90 | 4.00 | 27.00 | 0.0000 | *** |
| te(derivate tide, tide) | 3.00 | 3.00 | 14.30 | 0.0026 | ** |
| s(SST) | 3.31 | 4.03 | 48.60 | 0.0000 | *** |
| s(hour): cluster | 7.70 | 26.00 | 45.20 | 0.0000 | *** |
| s(depth) | 2.14 | 2.17 | 34.10 | 0.0000 | *** |
| s(distance) | 2.80 | 2.83 | 48.20 | 0.0000 | *** |
